# Plasma Membrane Pores Drive Inflammatory Cell Death

**DOI:** 10.3389/fcell.2020.00817

**Published:** 2020-08-21

**Authors:** Benedikt Kolbrink, Theresa Riebeling, Ulrich Kunzendorf, Stefan Krautwald

**Affiliations:** Department of Nephrology and Hypertension, University Hospital Schleswig-Holstein, Kiel, Germany

**Keywords:** necroptosis, pyroptosis, MLKL, GSDMD, regulated cell death, necroinflammation

## Abstract

Necroptosis and pyroptosis are two forms of regulated cell death. They are executed by the proteins mixed-lineage kinase domain-like (MLKL) and gasdermin D (GSDMD), respectively. Once activated by numerous pathways, these proteins form membrane pores that allow the influx and efflux of various ions, proteins, and water, ultimately resulting in the death of the cell. These modalities of cell death are considered highly inflammatory because of the release of inflammatory cytokines and damage-associated molecular patterns, and are thereby not only deleterious for the dying cell itself, but also its environment or the entire organism. The relevance for these processes has been observed in various physiological and pathophysiological conditions, ranging from viral and bacterial infections over autoimmune and chronic inflammatory diseases to ischemic organ damage. In recent years, initial *in vitro* experiments have shed light on a range of connections between necroptosis and pyroptosis. Initial *in vivo* studies also indicate that, in many disease models, these two forms of cell death cannot be considered individually, as they demonstrate a complex interaction. In this article, we provide an overview of the currently known structure, pathways of activation, and functions of MLKL and GSDMD. With emerging evidence for an interconnection between necroptosis and pyroptosis in not only *in vitro*, but also *in vivo* models of disease, we highlight in particular the clinical relevance of the crosslinks between these two forms of inflammatory cell death and their implications for novel therapeutic strategies in a variety of diseases.

## Introduction

In recent years, a variety of different forms of regulated cell death (RCD) has been discovered, and many of the underlying molecular mechanisms have been deciphered (Galluzzi et al., 2018; Tang et al., 2019). The permeabilization of the plasma membrane by pore-forming proteins plays a crucial role in only two distinct necrotic forms of RCD, namely necroptosis and pyroptosis. Both are considered inflammatory RCD, as the dying cells release miscellaneous damage-associated molecular patterns (DAMPs) and pro-inflammatory cytokines that induce profound inflammation of the surrounding tissue (Bergsbaken et al., 2009; Pasparakis and Vandenabeele, 2015). Apart from the obvious similarities, however, there are also subtle and less subtle differences, cross-links, and cooperation in the development of diseases between necroptosis and pyroptosis. Herein, we focus on the executing proteins mixed-lineage kinase domain-like (MLKL) and gasdermin D (GSDMD), and provide an overview of their known mechanisms of activation and membrane permeabilization and the clinical relevance of understanding their interconnections.

## Necroptosis and MLKL

Mixed-lineage kinase domain-like is the most terminal known indispensable effector protein of necroptosis. It is a 54 kDa protein that consists of an N-terminal four-helix bundle (4HB) and the C-terminal pseudokinase domain (PKD), which are connected by a two-helix linker brace (Petrie et al., 2017). In necroptosis, MLKL is activated by receptor-interacting serine/threonine protein kinase 3 (RIPK3) via phosphorylation of the residues T357/S358 in humans or S345 in mice (Sun et al., 2012; Murphy et al., 2013; Rodriguez et al., 2016). To date, three different activation pathways of MLKL in the context of necroptosis have been described: the first and best characterized begins by death receptor signaling via TNFR1, TRAIL or Fas ligand, and is mediated by RIPK1. The second pathway follows the activation of toll-like receptor (TLR) 3 and 4 and is mediated via the adaptor protein TRIF (also known as TICAM1), whereas the third characterized pathway follows activation of the cytosolic RNA sensor ZBP1 (also known as DAI). Common to all these pathways is that, in the absence or impairment of caspase function, the RHIM domain-containing proteins RIPK1, TRIF, or ZBP1 bind RIPK3 via their respective RHIM domains to form the so-called necrosome, which is followed by RIPK3 autophosphorylation and consecutive activation of MLKL (Weinlich et al., 2017; Shan et al., 2018; Malireddi et al., 2019). Whether necroptosis is dependent on the generation of mitochondrial reactive oxygen species (ROS) has also been discussed, but more recent data have challenged this hypothesis. The evidence for and against this idea is described in detail (Marshall and Baines, 2014). Recently, it was shown that, at least for RIPK1-dependent necroptosis, ROS enhance the activation of RIPK1 by autophosphorylation in a positive feedback loop (Zhang et al., 2017). However, the exact mechanism of how MLKL is phosphorylated by RIPK3 and hence activated differs among species and has not been resolved. In human cells, RIPK3 needs to bind MLKL via its PKD to phosphorylate and activate it (Sun et al., 2012; Petrie et al., 2018), whereas in murine cells, MLKL is not recruited to RIPK3, and phosphorylation alone is sufficient for inducing necroptosis (Murphy et al., 2013; Hildebrand et al., 2014; Jacobsen et al., 2016). For different vertebrates, it was shown recently that varying conformations of the PKD in particular play an important role in species-specific activation of MLKL (Davies et al., 2020).

The structure of the membrane pore formed by MLKL has not been resolved to date. It is generally agreed that phosphorylation of the PKD leads to a conformational change that exposes the 4HB, which results in MLKL oligomerization in the cytoplasm, traffic to the cell periphery via Golgi-microtubule-actin-dependent mechanisms and subsequent membrane translocation by binding phosphatidylinositol phosphates (PIPs), but there is disagreement about whether MLKL forms trimers, tetramers, or hexamers (Cai et al., 2014; Chen et al., 2014; Dondelinger et al., 2014; Wang H. et al., 2014; Samson et al., 2020). Recently, we visualized necroptotic cell death in the presence of polyethylene glycols (PEGs) of different sizes and were able to estimate that the membrane pores executing necroptosis had inner diameters of around 4 nm (Ros et al., 2017). Once inserted into the plasma membrane, MLKL oligomers increase membrane permeability to cations, which leads to calcium and sodium influx and potassium efflux from the cell along the concentration gradients. This is followed by the influx of water into the cell along the osmotic gradient, and results in a unique morphology of the dying necroptotic cell, that is described as explosion-like (Chen et al., 2016; Xia et al., 2016).

The relevance for necroptosis has been shown in the pathophysiology of various diseases, both infectious and non-infectious. From a historical perspective, the concept of necroptosis was originally discovered in that small molecules, the so-called necrostatins, could suppress necrotic cell death in certain settings (Degterev et al., 2005). The next step was the discovery of RIPK1 as the target molecule of necrostatins and the subsequent identification of the other proteins involved in necroptosis, which then enabled investigations in knockout animals and animal models of specific diseases (Degterev et al., 2008; Silke et al., 2015). In an infectious context, necroptosis is a double-edged sword. It is of particular importance in the defense against viral pathogens and intracellular bacteria, although the mechanisms vary and have not been definitively elucidated. For specific infections, necroptosis has positive effects on the removal of pathogens, as shown in murine models of herpes simplex virus, cytomegalovirus, vaccinia virus, and influenza A virus (Cho et al., 2009; Upton et al., 2012; Wang X. et al., 2014; Nogusa et al., 2016). Remarkably, the clearance of Listeria monocytogenes also relies on RIPK3 and MLKL, which in this case does not lead to the death of the host cell, but inhibits bacterial growth by direct binding of MLKL to the bacteria (Sai et al., 2019). However, there are adverse effects of necroptosis in infections. In mouse models of infection with *Salmonella enterica* or *Staphylococcus aureus*, triggering of necroptosis by the pathogens is a deleterious process for the host (Robinson et al., 2012; Kitur et al., 2015). Apart from infections, necroptosis also has a function in sterile pathologies. Genetic deletion or pharmacological inhibition of RIPK1/3 and MLKL improve the outcome in models of systemic inflammatory response syndrome (Zelic et al., 2018; Moerke et al., 2019), and ischemia-reperfusion injury of the kidney (Müller et al., 2017), brain (Naito et al., 2020), and heart (Luedde et al., 2014). The RIPK3-MLKL axis is also involved in crystallopathies induced by cytotoxic crystals (Mulay et al., 2016). Cancer metastasis also depends on necroptosis (Strilic et al., 2016). As shown recently, necroptosis also plays a role in inflammatory skin diseases. In a mouse model of psoriasis, it was shown that inhibition of the RIPK1-RIPK3-MLKL axis has a protective effect (Duan et al., 2020), and that, in the absence of RIPK1 in keratinocytes, ZBP1-induced necroptosis leads to massive inflammation of the skin (Devos et al., 2020). The fact that two very different stimuli can trigger necroptosis via different pathways underlines the complexity of this topic, especially with regard to possible therapeutic interventions. A function of the necroptosis signaling pathway has also been demonstrated in a broad range of other disease models, which is reviewed elsewhere in greater detail (Weinlich et al., 2017). Most recently, certain homozygous mutations in the *Mlkl* gene that lead to a lethal inflammatory phenotype in mice were described for the first time. For unknown reasons, this phenotype seems to be caused by hyperinflammation of the mediastinum and salivary glands. In heterozygous human individuals, however, these mutations are associated with increased incidence of the autoimmune disease chronic recurrent multifocal osteomyelitis (Hildebrand et al., 2020). Taken together, there is ever growing evidence for necroptosis as a promising target for therapeutic efforts in a multitude of highly relevant diseases, although the mechanisms require further clarification.

## Pyroptosis and GSDMD

Pyroptosis is executed by the proteins of the gasdermin family. This chapter focuses on GSDMD, as it is so far the best characterized of these homologous pore-forming proteins and is conserved in humans and mice. GSDMD is a cytosolic 53 kDa protein expressed in immune cells and epithelia, and consists of a cytotoxic N-terminal domain (GSDMD-N), a linker region, and an inhibitory C-terminal domain (GSDMD-C) (Kuang et al., 2017). Although structurally similar at first glance, GSDMD is, in contrast to MLKL, not activated via phosphorylation but through proteolytic cleavage (Kayagaki et al., 2015; Shi et al., 2015). Two major pathways of GSDMD activation have been described. The so-called canonical pathway is dependent on the formation of multiprotein complexes called inflammasomes. Inflammasomes are formed by cytosolic pattern recognition receptors (PRRs) termed nucleotide-binding oligomerization domain (NOD) and leucine-rich repeat (LRR) receptors (NLRs), absent in melanoma 2 (AIM2)-like receptors, and retinoic acid-inducible gene I (RIG I)-like receptors. One of the best studied of these is the NLR protein 3 (NLRP3) inflammasome (Broz and Dixit, 2016). Canonical activation of the NLRP3 inflammasome requires two steps: in the first priming step, DAMPs or pathogen-associated molecular patterns (PAMPs) activate various PRRs such as TLRs, which leads to upregulation in the synthesis of critical inflammasome components such as NLRP3 itself, apoptosis-associated speck-like protein containing a caspase recruitment domain (ASC), procaspase-1, and the inactive precursor cytokines pro-interleukin (IL)-1β and pro-IL-18. A second step is needed for inflammasome activation. A critical element of this activation step are changes in intracellular potassium and chloride concentrations, the function of which we have gained better understanding in recent years. To assemble the NLRP3 inflammasome, cytosolic ASC oligomers or specks must first be formed, and this process is dependent on chloride efflux from the cell. This chloride efflux relies on the translocation of chloride intracellular channels (CLICs) to the plasma membrane and is an indispensable upstream event of NLRP3 inflammasome activation (Tang et al., 2017; Green et al., 2018). Besides the CLICs, chloride outflow from the volume-regulated anion channel (VRAC) is also described in this context, which can be inhibited by certain non-steroidal anti-inflammatory drugs (Daniels et al., 2016). In addition to chloride, potassium efflux is also necessary for inflammasome activation, and can be triggered by various mechanisms. Ionophores and bacterial toxins such as nigericin or streptolysin O can facilitate potassium efflux, making the NLRP3 inflammasome a sensor of deleterious microorganisms (Petrilli et al., 2007; Greaney et al., 2015). In addition, cell damage in the surrounding tissue, through which ATP enters the extracellular space, can be sensed by the ligand-gated purinergic P2X7 ion channel receptor (P2X7R). Thus, the cell membrane is permeabilized for cations, and potassium efflux becomes possible (Alves et al., 2014). The potassium efflux then leads to the association of NIMA-related kinase 7 (NEK7) with NLRP3 and subsequent inflammasome activation, which is followed by the cleavage of procaspase-1 into its active form and the processing and release of IL-1β and IL-18 (Munoz-Planillo et al., 2013; He et al., 2016). As with the execution of necroptosis, the influence of disturbances in the redox homeostasis of the cell and ROS generation from several sources on NLRP3 inflammasome activation has also been discussed, although the underlying mechanisms are not fully understood (reviewed in Abais et al., 2015). The further details of canonical inflammasome activation exceed the scope of this review, and are described excellently elsewhere (Schroder and Tschopp, 2010; Broz and Dixit, 2016). Besides the canonical pathway, there are other means of initiating pyroptosis. The non-canonical pathway is a “shortcut” for the activating GSDMD. Upon sensing intracellular bacterial lipopolysaccharide (LPS), the inflammatory caspases-4/5 (in humans) and caspase-11 (in mice) can directly cleave GSDMD and initiate pyroptosis (Shi et al., 2014). GSDMD can also be activated directly by caspase-8 when transforming growth factor β-activated kinase 1 (TAK1), a central regulator of cell death and survival in a variety of settings, is inhibited, as described in the case of *Yersinia pseudotuberculosis* infection (Orning et al., 2018; Sarhan et al., 2018). Apart from this, GSDMD activation by the proteases cathepsin G and neutrophil elastase has been reported in myeloid cells (Sollberger et al., 2018; Burgener et al., 2019).

Common to all these pathways initiating pyroptosis is the proteolytic cleavage of GSDMD in the linker region, which releases GSDMD-N from its autoinhibition by GSDMD-C. GSDMD-N forms oligomers that create membrane pores. Therefore, GSDMD-N translocates exclusively to the inner leaflet of cell membranes by binding PIPs, phosphatidic acid, and phosphatidylserine (Ding et al., 2016; Liu et al., 2016). The nanostructure of the gasdermin pore has been described for mouse gasdermin A3 via cryo-electron microscopy. It appears to be a 26–28-fold symmetric, anti-parallel β-barrel pore with an inner diameter of 18 nm (Ruan et al., 2018). The size of the human GSDMD-N pore is estimated to be 10–20 nm (Ding et al., 2016; Sborgi et al., 2016). GSDMD-N also has the ability to bind cardiolipin, and there are reports of gasdermin-mediated permeabilization of mitochondria contributing to pyroptotic cell death (de Vasconcelos et al., 2019; Rogers et al., 2019). The GSDMD-N pore is structurally and functionally different from the MLKL pore. Intracellular osmolality is unchanged during pyroptosis, as the GSDMD-N pore is unselective and allows free diffusion of ions and water as well as the release of processed cytokines, the first and foremost being IL-1β and IL-18 (Heilig et al., 2018). This difference also finds its expression in the varying morphology between necroptosis and pyroptosis. In pyroptosis, there is no swelling or explosive rupture of the cells, but a flattening of the cytoplasm, as cytoplasmic fluid leaks through the pores (Chen et al., 2016).

Analogous to necroptosis, most of the understanding of the (patho-)physiological role of pyroptosis has been generated through the utilization of animal models deficient for the inflammatory caspases, inflammasome components, IL-1 and IL-18, or GSDMD. Countering infections is doubtless one of the most crucial roles of pyroptosis. The clearance of viral, bacterial, fungal, and protozoan infections relies on it to differing extents, depending on the underlying microorganism and context (Man et al., 2017). Direct lysis of bacteria through its ability to bind cardiolipin in the bacterial cell wall has been described for GSDMD-N (Liu et al., 2016). Counterintuitively, and all the more surprising, is the fact that *Gsdmd*-deficient mice showed improved survival in a model of infectious peritonitis by *Escherichia coli*, as *Gsdmd* deficiency in this case prolonged neutrophil survival and therefore improved bacterial clearance (Kambara et al., 2018). These findings once again underscore the complexity of RCD in disease. Besides infectious diseases, there is also evidence that pyroptosis has a role in sterile diseases, and these largely overlap those in which necroptosis has also shown relevance, such as in ischemia-reperfusion injury of the heart (Qiu et al., 2017), brain (Yang F. et al., 2014), and kidney (Yang J.R. et al., 2014), crystallopathies (Ludwig-Portugall et al., 2016; Goldberg et al., 2017), Alzheimer’s disease (Han et al., 2020), and other neurodegenerative pathologies (McKenzie et al., 2020) and a multitude of other inflammatory diseases (Goldberg et al., 2017). Of particular note in this context is the recent discovery that ticagrelor – a substance usually administered in coronary heart disease due to its inhibitory properties on platelet aggregation – also inhibits the NLRP3 inflammasome and therefore pyroptosis by blocking the CLIC-dependent chloride efflux (Huang et al., 2020). This interesting double effect could explain part of the good efficacy after coronary interventions. However, much of this research has been conducted through genetic ablation or pharmacological inhibition of the NLRP3 inflammasome, and requires future testing of the models in specific *Gsdmd* knockout mice to clearly discriminate the exact contribution of pyroptosis itself from other inflammasome-driven effects.

## Crosslinks Between Necroptosis and Pyroptosis

Although it was and still is necessary to consider the different forms of RCD in isolation to decipher their molecular pathways, there is emerging evidence for combined action of specific forms of RCD in particular diseases. We ourselves first demonstrated that ischemia-reperfusion injury of the kidney was not only mediated by necroptosis (Linkermann et al., 2012), but also by the combination of necroptosis and ferroptosis, an iron-dependent form of regulated cell death driven by phospholipid peroxidation (Müller et al., 2017), and was greatly ameliorated by combined targeting of necroptosis and the RCD cyclophilin D-mediated mitochondrial permeability transition (Linkermann et al., 2013). Other authors have reported the cooperation of apoptosis and necroptosis in the pathology of ischemic stroke (Naito et al., 2020).

**FIGURE 1:**
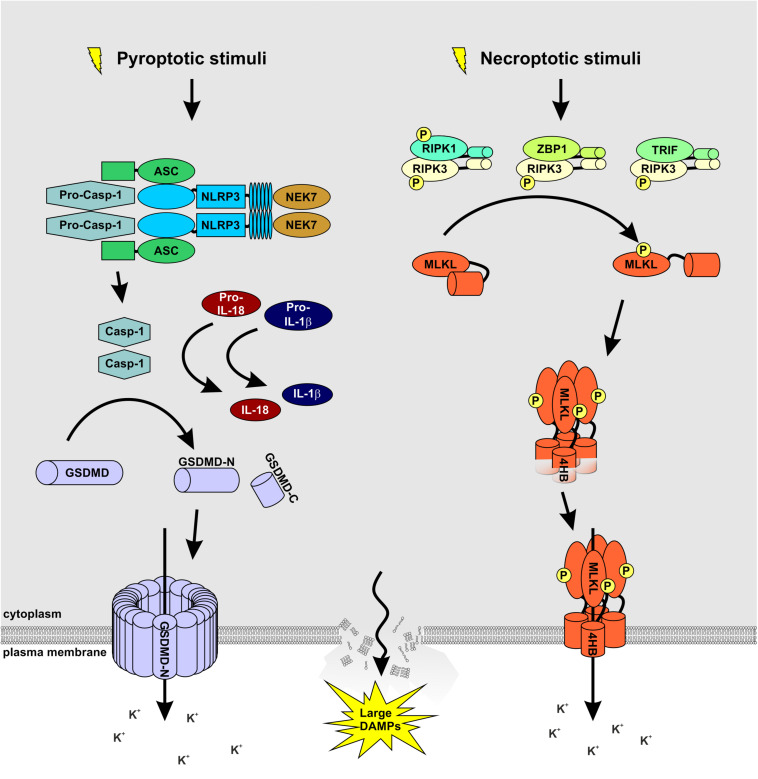
MLKL and GSDMD: partners in crime. A wide array of extracellular stimuli such as bacteria, viruses, and toxins serve as cellular stressors that activate NLRP3. The latter activates caspase-1 via the adaptor protein ASC. Caspase-1 processes and activates IL-1β and IL-18 and cleaves GSDMD to release the membrane pore-forming GSDMD-N domain. Necroptosis is induced by ligand binding to TNF family death domain receptors, PRRs, and virus sensors, which promote the assembly of RIPK3-MLKL complex. RIPK3-mediated phosphorylation of MLKL results in MLKL oligomerization followed by translocation to the plasma membrane to induce membrane rupture. Notably, both signaling pathways cause potassium efflux, accompanied by water influx, cell swelling, and osmotic lysis, propagating the pro-inflammatory responses triggered by NEK7 binding to NLRP3.

Recently, such an interaction has also become apparent between necroptosis and pyroptosis. Initial *in vitro* studies have shown RIPK3-dependent processing of IL-1 in the absence of the inhibitors of apoptosis proteins (IAPs), which play a critical role in several RCD pathways and human disease (Vince et al., 2012). The refined and complex mechanism of this process has been elucidated in further excellent research (Yabal et al., 2014; Lawlor et al., 2017). At the same time, a role for MLKL has been brought into play, and inflammasome activation dependent on the RIPK1-RIPK3-MLKL axis in dendritic cells has been reported (Kang et al., 2013) as well as MLKL-dependent and -independent RIPK3-mediated inflammasome activation (Kang et al., 2015; Lawlor et al., 2015). In 2017, it was shown that the activation of MLKL is sufficient for inflammasome assembly and the release of mature IL-1β even in absence of GSDMD, a prime example of how different forms of RCD can compensate for each other (Gutierrez et al., 2017). Only weeks later, a second group similarly reported a cell-intrinsic activation of the NLRP3 inflammasome by MLKL during the induction of necroptotic cell death (Conos et al., 2017). These two studies also clearly identified the underlying mechanism: MLKL-mediated potassium efflux from the cell is the indispensable activation step analogous to the canonical pathway of inflammasome activation (Figure 1).

The first strong indications of an *in vivo* connection have been reported in a mouse model of *S. aureus* pneumonia, in which the inhibition of MLKL-dependent necroptosis decreased caspase-1 activation and IL-1β processing (Kitur et al., 2015). Second, in a mouse model of oral infection with *Salmonella enterica serovar Typhimurium*, MLKL-dependent initiation of pyroptosis in the caecum was needed for effective clearance of the pathogen, whereas *Mlkl*-deficient mice died significantly faster than their wild-type counterparts (Yu et al., 2018). Especially interesting in this case is the fact that Yu and colleagues showed, via the generation of bone marrow chimeras, that MLKL and therefore pyroptosis in non-hematopoietic cells conferred the protective effect against infection. Up to that point, a connection between MLKL and inflammasome activation had, to our knowledge, only been reported in cells of myeloid heritage. Another noteworthy connection between necroptosis and pyroptosis was found in the effect of the small molecule necrosulfonamide (NSA). NSA was initially described in a groundbreaking work by Xiaodong Wang’s group (Sun et al., 2012). It covalently binds to human MLKL via the Cys86 residue, inhibiting its activation by RIPK3 and consequently necroptosis. Surprisingly, NSA is also capable of binding GSDMD via Cys191, inhibiting pyroptotic cell death not only *in vitro* in human and mouse cell lines but also *in vivo* in murine sepsis models (Rathkey et al., 2018). Most recently, a mouse model of combined *Ripk3/Gsdmd* and *Mlkl/Gsdmd* knockout was reported. The double knockout showed significantly better protection in a mouse model of polymicrobial sepsis, and by generating bone marrow chimeras, the relevance of hematopoietic as well as non-hematopoietic cells in the model could be documented (Chen et al., 2020). Latterly, a connection between the three different forms of RCD pyroptosis, apoptosis, and necroptosis (PAN) has been shown *in vitro*. Evidence that these three forms of cell death may be linked has been found in specific settings. In influenza A virus infection, ZBP1 is a central regulator of PAN (Kuriakose et al., 2016; Kesavardhana et al., 2017). Additionally, the genetic- or pathogen-induced loss of TAK1 can also trigger pyroptosis, apoptosis, and necroptosis (Malireddi et al., 2018; Orning et al., 2018; Podder et al., 2019). This threefold activation of the cell death pathways was eventually termed PANoptosis (Malireddi et al., 2019). The most recent research in this field shows that this process is under the control of a master protein complex termed the PANoptosome (Christgen et al., 2020). Despite all this excellent work, our mechanistic understanding of these processes *in vivo* remains very vague. Based on the available data, it can be assumed that necroptosis and pyroptosis in particular play a common role not only in sepsis, but in a variety of other diseases, and we eagerly await further research in this area.

## Conclusion and Clinical Relevance

Over the last few years, it has become clear that the forms of inflammatory RCD have a critical position in the pathophysiology of almost all known diseases. Membrane pores and ion fluxes play a central role in these cell death processes and are already promising targets for therapeutic approaches. However, it is becoming equally clear that different cell death pathways have context-dependent positive or negative impacts in that they can compensate for each other and have synergistic or antagonistic effects. Therefore, their therapeutic applications in humans must be tested with great care, and it must be clarified beyond doubt whether and to what extent the different forms of RCD are advantageous or disadvantageous in the respective disease and how they are interconnected. A further complicating factor is that an attempt at therapeutic intervention in one pathway may unexpectedly have an impact on others. Besides testing combined gene knockouts in disease models, it would be of special interest to see whether a combined pharmacological approach of inhibiting necroptosis and pyroptosis would generate the same effect. Future efforts will have to focus on these interactions, as intervening in these pathways promises new and better treatment options for many previously poorly treatable diseases, including stroke, myocardial infarction, acute and chronic kidney failure, sepsis, systemic inflammatory response syndrome, and cancer.

## Author Contributions

UK contributed valuable ideas. TR generated the figure. BK and SK wrote the manuscript with input from all authors.

## Conflict of Interest

The authors declare that the research was conducted in the absence of any commercial or financial relationships that could be construed as a potential conflict of interest.
